# Supplementary material for: ES-RED (Early Seizure Recurrence in the Emergency Department) Calculator: A Triage Tool for Seizure Patients
Source: J Clin Med. 2022 Jun 22;11(13):3598. doi: 10.3390/jcm11133598 (PMC9267812; doi:10.3390/jcm11133598)
Supplement: Supplementary file 1 [file jcm-11-03598-s001.zip › Supplementary tables.pdf]

Table S1: LASSO analyses of variables associated with ES-RED in the derivation cohort

	β at minimal lambda	β at one standard error lambda
Age (per 1 year increase)	0.111	0.081
Prior anti-seizure medication ≥ 2 (vs. no ASM or 1 ASM)	0.278	0.113
Seizure count within 24h ≥ 2 (vs. less than 2 seizures)	0.490	0.340
Systolic blood pressure (per 1mmHg increase)	0.320	0.121
Glasgow coma scale (per 1 point increase)	-0.076	-0.011
Haemoglobin (per 1mg/dL increase)	-0.281	-0.085
Glucose (per 1mg/dL increase)	0.171	0.137
Albumin (per 1mg/dL increase)	NA	NA
Uric acid (per 1mg/dL increase)	-0.187	-0.053
K (per 1mmol/L increase)	-0.126	NA
Cl (per 1mmol/L increase)	NA	NA
Lactic acid (per 1mmol/L increase)	0.389	0.216
Any symptomatic cause (vs. neither acute nor remote symptomatic cause)	NA	NA

LASSO, Least Absolute Shrinkage and Selection Operator; ES-RED, Early Seizure Recurrence in the Emergency Department; ASM, antiseizure medication;

NA, not applicable.

Table S2: Comparison of variables between the derivation and validation cohort

	Derivation cohort (N=461)	Validation cohort (N=227)	p-value
**Demographics**			
Age	44 [26–57.5]	39 [26–59]	0.516
Sex, female	181 (39.3%)	87 (38.3%)	0.813
**History of medical disease**			0.519
None	309 (67.0%)	151 (66.5%)	
Diabetes/Hypertension/Dyslipidemia	48 (10.4%)	28 (12.3%)	
Liver	12 (2.6%)	7 (3.1%)	
Kidney	18 (3.9%)	13 (5.7%)	
Thyroid	6 (1.3%)	1 (0.4%)	
Cancer	21 (4.6%)	9 (4.0%)	
Cardiovascular	23 (5.0%)	5 (2.2%)	
Pulmonolgic/rheumatologic/other	24 (5.2%)	13 (5.7%)	
**History of neurological disease**			0.884
None	137 (29.7%)	62 (27.3%)	
Epilepsy	172 (37.3%)	95 (41.9%)	
Stroke	75 (16.3%)	33 (14.5%)	
Brain tumor	14 (3.0%)	8 (3.5%)	
Infection/inflammation	7 (1.5%)	4 (1.8%)	
Other	56 (12.1%)	25 (11.0%)	
**Seizure characteristics**			
Seizure semiology			0.003
Bilateral impaired awareness motor seizure only	352 (76.4%)	156 (68.7%)	
Focal feature	57 (12.4%)	51 (22.5%)	
Unwitnessed	52 (11.3%)	20 (8.8%)	
Seizure duration			0.145
< 3 mins	182 (39.5%)	103 (45.4%)	
≥ 3 mins	211 (45.8%)	86 (37.9%)	
Unknown	68 (14.8%)	38 (16.7%)	
Seizure count within 24h	1 [1–1]	1 [1–2]	0.018
Seizure count within 24h ≥ 2	87 (18.9%)	61 (26.9%)	0.016
Triggering factor			
Alcohol-related	68 (14.8%)	22 (9.7%)	0.064
Sleep deprivation	116 (25.2%)	50 (22.0%)	0.366
Previous seizure history	264 (57.3%)	130 (57.3%)	>0.999
Number of prior anti-seizure medication			0.739
no/unknown	278 (60.3%)	142 (62.6%)	
1	99 (21.5%)	43 (18.9%)	

≥2	84 (18.2%)	42 (18.5%)	
Prior anti-seizure medication ≥ 2	84 (18.2%)	42 (18.5%)	0.929
**Vital signs and exam**			
Systolic blood pressure, mmHg	126 [110–144]	128 [110–147]	0.409
Diastolic blood pressure, mmHg	78 [69–89]	80 [70–90]	0.199
Pulse rate, beats per minute	86 [78–100]	88 [78–102]	0.434
Body temperature, ℃	36.7 [36.4–36.9]	36.6 [36.3–36.9]	0.083
Glasgow coma score	15 [15–15]	15 [14–15]	0.638
Neurologic examination			0.986
Normal	350 (75.9%)	172 (75.8%)	
Focal abnormal	27 (5.9%)	14 (6.2%)	
Diffuse abnormal	84 (18.2%)	41 (18.1%)	
**Laboratory findings**			
White blood cell, $10^3/\mu\ell$	8.05 [6.3–10.7]	7.9 [6.0–10.35]	0.537
Haemoglobin, g/dl	13.6 [12.4–14.875]	13.7 [12.5–15.1]	0.251
Platelet, $10^3/\mu\ell$	225 [182–271]	226 [177.5–271.5]	0.967
Erythrocyte sedimentation rate, mm/hr	7.5 [2–17.25]	6 [3–12]	0.370
C-reactive protein, mg/dL	0.1 [0.03–0.4]	0.095 [0.04–0.3075]	0.768
Glucose, mg/dL	111 [97.5–133]	108 [96–139.75]	>0.999
Albumin, g/dL	4.5 [4.2–4.8]	4.6 [4.3–4.8]	0.155
Uric acid, mg/dL	6.5 [4.55–8.8]	6.1 [4.8–8.4]	0.961
Creatine kinase, U/L	125.5 [82.25–218.5]	129 [83.5–228.5]	0.934
Blood urea nitrogen, mg/dL	11.65 [9.325–14.8]	11.8 [9.05–14.85]	0.693
Creatinine, mg/dL	0.815 [0.69–0.97]	0.85 [0.68–1.025]	0.158
Na, mmol/L	140 [138–141]	139 [137–141]	0.140
K, mmol/L	4.0 [3.725–4.20]	4.1 [3.8–4.3]	0.006
Cl, mmol/L	101 [99–103]	101 [98–103]	0.412
Ca, mg/dL	5.04 [4.6–9.2]	5.445 [4.7–9.2]	0.348
Mg, mg/dL	2.1 [2.0–2.3]	2.2 [2.0–2.3]	0.086
Ammonia, umol/L	29 [19–43.5]	26 [16–41]	0.273
Lactate, mmol/L	2.575 [1.6275–4.645]	2.355 [1.4675–4.53]	0.197
pH	7.391 [7.348–7.421]	7.3835 [7.3518–7.4173]	0.727
Base Excess, mmol/L	-1.95 [-4.2 to -0.075]	-1.65 [-4.4 to 0]	0.510
Bicarbonate, mmol/L	22.2 [19.875–24.125]	22.3 [19.8–24.625]	0.634
pCO2, mmHg	36.9 [33.15–41]	36.85 [32.65–41.65]	0.774
**Diagnostic evaluation**			
Implemented CT scan			0.850
Normal	213 (46.2%)	101 (44.5%)	
Abnormal	137 (29.7%)	67 (29.5%)	

Not done	111 (24.1%)	59 (26.0%)	
Implemented MRI scan			0.511
Normal	89 (19.3%)	36 (15.9%)	
Abnormal	58 (12.6%)	32 (14.1%)	
Not done	314 (68.1%)	159 (70.0%)	
Implemented EEG			0.057
Normal	68 (14.75%)	20 (8.81%)	
Abnormal	115 (24.95%)	53 (23.35%)	
Not done	278 (60.3%)	154 (67.84%)	
**Etiology**			
Acute symptomatic	28 (6.1%)	23 (10.1%)	0.056
Remote symptomatic	87 (18.9%)	43 (18.9%)	0.982
Any symptomatic	112 (24.3%)	64 (28.2%)	0.270
**IV benzodiazepine in ED**	134 (29.1%)	61 (26.9%)	0.548

Values are represented as median [interquartile range] or number (percentage). ES-RED, Early Seizure Recurrence in the Emergency Department; CT, computed tomography; MRI, magnetic resonance imaging; EEG, electroencephalogram; IV, intravenous; ED, emergency department.
