# Supplementary figures and images for: ES-RED (Early Seizure Recurrence in the Emergency Department) Calculator: A Triage Tool for Seizure Patients
Source: J Clin Med. 2022 Jun 22;11(13):3598. doi: 10.3390/jcm11133598 (PMC9267812; doi:10.3390/jcm11133598)

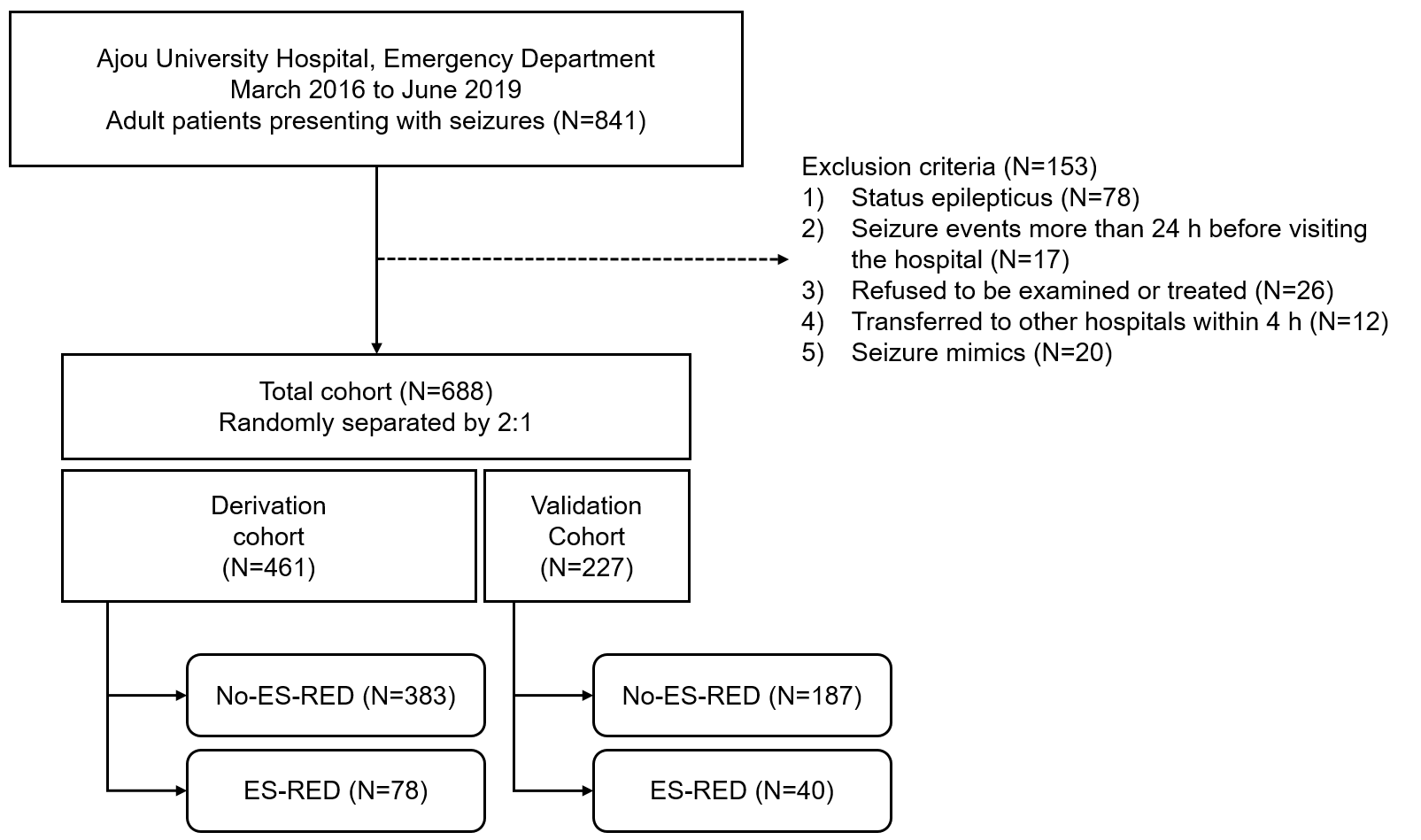

Supplement: Supplementary file 1 [file jcm-11-03598-s001.zip › Figure S1.tif]
